# Supplementary material for: The interactome of intact mitochondria by cross-linking mass spectrometry provides evidence for coexisting respiratory supercomplexes
Source: Mol Cell Proteomics. 2017 Dec 8;17(2):216–32. doi: 10.1074/mcp.RA117.000470 (PMC5795388; doi:10.1074/mcp.RA117.000470)
Supplement: Supplemental Data [file supp_RA117.000470_133922_0_supp_23978_fzffwf.zip › spectra_annotation/mito_spectra_annotation/189-1-3-1-4-1.pdf]

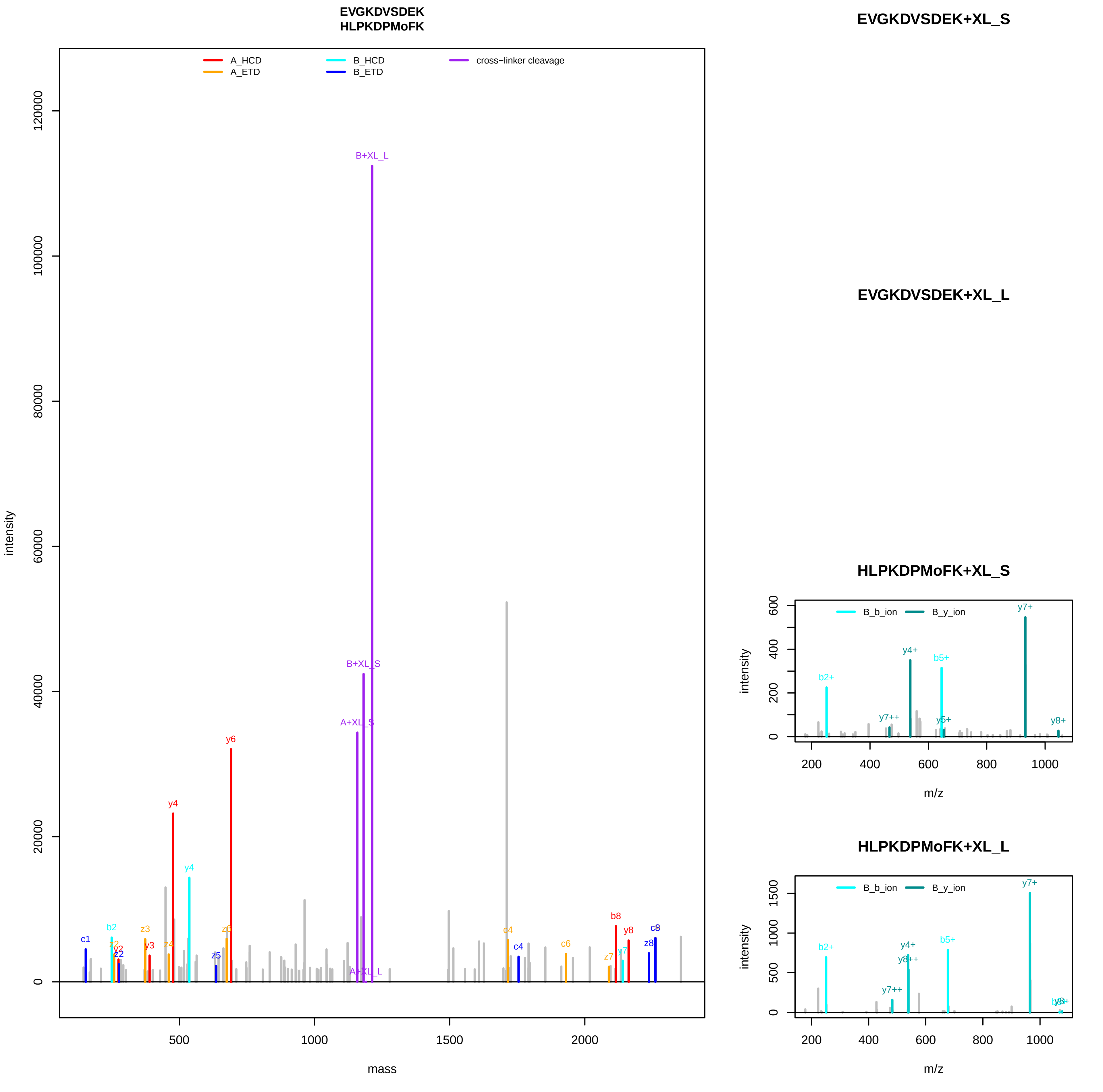
EVGKDVSDEK
HLPKDPMoFK
A_HCD
A_ETD
B_HCD
B_ETD
cross−linker cleavage
B+XL_L
B+XL_S
A+XL_S
A+XL_L
y6
y4
y4
b8
y8
c8
z8
c1
b2
z2
y2
z2
z3
y3
z4
z5
z6
c4
c4
c6
z7
y7
intensity
mass
0
20000
40000
60000
80000
100000
120000
500
1000
1500
2000
EVGKDVSDEK+XL_S
EVGKDVSDEK+XL_L
HLPKDPMoFK+XL_S
B_b_ion
B_y_ion
y7+
y4+
b5+
b2+
y7++
y5+
y8+
intensity
m/z
0
200
400
600
800
1000
HLPKDPMoFK+XL_L
B_b_ion
B_y_ion
y7+
b5+
y4+
y8++
b2+
y7++
y8+
intensity
m/z
0
500
1000
1500
200
400
600
800
1000
